# Supplementary material for: “What’s the best way to document information concerning psychiatric patients? I just don’t know”—A qualitative study about recording psychiatric patients notes in the era of electronic health records
Source: PLoS One. 2022 Mar 3;17(3):e0264255. doi: 10.1371/journal.pone.0264255 (PMC8893630; doi:10.1371/journal.pone.0264255)
Supplement: Attachment — Submitted filename: response to editor.docx [file pone.0264255.s002.docx]

Dear Dr. Maria Berghs,

Many thanks for your email with comments and suggestions. The authors have carefully considered the comments and tried our best to address every one of them.
Below we provide the point-by-point responses. All modifications in the manuscript have
been highlighted in red.

Best regards,

Olga Chivilgina

*The article is not of publishable standard. This has nothing to do with the data nor methods but the presentation of the findings and formatting. So, I hope you will attend to these issues. One reviewer has advised to accept but before we can accept it, I would like you to ensure that, for example, when presenting your findings that you signpost to the reader what these are.. This is why the headings did not make sense for the second reviewer. who requested major revisions. So, I would insert a short paragraph before the presentation of the findings, signposting the main themes (your headings) and explain that you will discuss these in turn. The results section with table seems to correspond to your methods (how many people participated in your research/who/sample) and the findings are about your themes that you found in your data analysis. The way it is currently laid out is confusing to the reader.*

Thank you for pointing this out. We have inserted a paragraph before the results section:

“The results section is structured in a following way: First, positive aspects of EHR are presented. Overall, while participants acknowledged positive aspects of EHR, they raised multiple concerns regarding psychiatric patients including: 1) issues related to the accessibility of personal data in EHR, 2) multidisciplinary teams access to confidential data, and 3) writing reports and notes in EHR. Then, we observe psychiatry-related factors, which aggravate the complexity of the problem of data sharing in EHR: these are stigma in mental health, and moral dilemmas related to mental health specialists who decide either to preserve data confidentiality, maintaining patient trust, or to share it with other healthcare specialists. In light of these various challenges, participants suggested solutions on how to document in EHR and how to preserve data confidentiality, which we summarize in the tables (Table 3 and 4). At the end of the section, we provide the responses of the participants on the hypothetical case, which was mentioned in the methods section”.

*I would also encourage you to look at some other papers in the journal and see what the conventions are when sharing quotations and how they signpost. I would advise you to signpost more and also present quotations likewise so that it is the voices of your participants that are highlighted: For example, here the voice is first: 
“Many patients are telling me "I'm going to say that. But please don't write it. Or please don't make any notes of it". They wanted to keep it between the two of us. I think because there is a legitimate fear” (P14, HP,F)*

Thanks for this important comment. We have changed the quotation style.

*At the moment , in places, your quotations are lists rather than ethical analysis of what people are saying. Could you use fewer quotations or format so we can see them clearer? The section on Trust and Transparency is good.*

Indeed, some quotations seem to be bulky. We have shortened (as much as possible) or deleted some quotations.

*Again, before your discussion it might be useful to signpost to the reader what the main points will be that you are discussing.*

We added a small paragraph before the discussion section:

“In this section, we discuss EHR in a context of the Swiss healthcare system and analyze the problems mentioned be participants. Then we outline the ethical issues associated with sharing sensitive data in the electronic patient dossier (EPD)”.

*Please check your first reference and that you do not have to mention an author?*

Unfortunately, the name of the journalist was not published online.

*The conflicts of interests section goes at the end of an article and not before the conclusion.*

According to your comment, we have moved the section “conflicts of interests” at the end of the article.
